# MSDSANet: Multimodal Emotion Recognition Based on Multi-Stream Network and Dual-Scale Attention Network Feature Representation

**DOI:** 10.3390/s25072029

**Published:** 2025-03-24

**Authors:** Weitong Sun, Xingya Yan, Yuping Su, Gaihua Wang, Yumei Zhang

**Affiliations:** 1 School of Digital Art, Xi’an University of Posts & Telecommunications, Xi’an 710061, China; 2 Key Laboratory of Intelligent Media in Shaanxi Province Colleges and Universities, Xi’an 710061, China; 3 Key Laboratory of Intelligent Computing and Service Technology for Folk Song, Ministry of Culture and Tourism, Xi’an 710062, China; 4 School of Computer Science, Shaanxi Normal University, Xi’an 710062, China

**Keywords:** multimodal, multi-scale, attention, emotion recognition

## Abstract

Aiming at the shortcomings of EEG emotion recognition models in feature representation granularity and spatiotemporal dependence modeling, a multimodal emotion recognition model integrating multi-scale feature representation and attention mechanism is proposed. The model consists of a feature extraction module, feature fusion module, and classification module. The feature extraction module includes a multi-stream network module for extracting shallow EEG features and a dual-scale attention module for extracting shallow EOG features. The multi-scale and multi-granularity feature fusion improves the richness and discriminability of multimodal feature representation. Experimental results on two datasets show that the proposed model outperforms the existing model.

## 1. Introduction

Emotion is a complex subjective experience intertwined with cognition, expression, motivation, and physiological factors, which constitute the key physiological and psychological states of the human body and are closely related to an individual’s behavioral motivation [1]. Emotions not only reflect the underlying motivation and consciousness behind human behavior but also play a direct role in the construction and maintenance of interpersonal relationships, cognitive processes, decision-making, and work efficiency [2]. In today’s fast-paced life and high-pressure social competition environment, an increasing number of people are facing the problem of emotional regulation. Long-term immersion in negative emotions not only harms our physical and mental health, but also greatly reduces our quality of life and well-being. In view of this, it is particularly necessary to carry out research on emotion recognition and realize automatic recognition of emotion.

Based on the distinct sources of emotional information and recognition methodologies, emotions can be categorized into two primary types: external emotion and internal emotion. External emotion recognition is mainly used to infer the emotional state [3] of an individual through the observation and analysis of his or her external performance, which covers fluctuations in speech intonation [4], subtle facial expression fluctuations [5], and overall characteristics of individual behavior patterns [6]. Internal emotion recognition focuses on the analysis of an individual’s internal physiological signals to determine their emotional state, including heart rate [7], respiratory rate, electroencephalogram (EEG) [8], skin conductivity, and electrooculogram (EOG) [9]. Each method has its own unique advantages. External behavioral signals have more advantages in terms of the convenience of data collection, while internal physiological signals are valued for their objectivity and reliability in emotional expression [10]. Therefore, emotion recognition methods based on internal physiological signals have gradually become mainstream in research because they can truly reflect emotions. Electroencephalography (EEG) plays an important role in the analysis of brain science and is one of the most promising emotion recognition technologies.

With excellent feature extraction and pattern recognition capabilities, deep learning can deeply mine the complex emotional features hidden in EEG signals to achieve accurate feature extraction and pattern recognition, which has attracted many researchers to carry out research on EEG emotion recognition. Liang et al. innovatively applied the unsupervised decoding method to EEG decoding, aiming to achieve efficient EEG feature description and integration [11]. Song et al. proposed an EEG-Conformer network architecture for effectively coupling local and global features in EEG signals [12]. The 4D-CRNN model uses a CNN to accurately capture and extract the spatial distribution and frequency component information of EEG signals and processes the time dimension information through an LSTM [13]. EmotionNet uses three-dimensional convolution operations to simultaneously process information in both the spatial and temporal dimensions of EEG signals [14]. Song et al. developed a DGCNN model that improved the recognition accuracy to 90.4% by autonomously learning the adjacency matrix [15]. Zhang et al. developed a GCB-Net model that captures complex spatial and temporal correlations of brain signals [16]. Song et al. designed an instance adaptive graph neural network that can dynamically construct and adjust the graph structure to accurately capture the functional connections between various brain regions and their dynamic adjustment with the change of emotional state [17]. The MAS-DGAT-Net model combines the advantages of a graph attention network and dynamic learning to comprehensively extract spatiotemporal information from EEG signals [18]. Parallel spatiotemporal frequency neural networks can more comprehensively reveal the complex mechanisms of the brain in the process of emotion processing by synchronously extracting and integrating the spatiotemporal frequency characteristics of EEG signals [19]. By fusing time-flow shared convolutions and time-fine spatiotemporal convolutions, Lu et al. successfully constructed a model for the efficient extraction of spatiotemporal dimensions of latent affective information [20].

The continuous innovation and expansion in the field of emotion computing has promoted the application of electroencephalography and various physiological signals in emotion recognition tasks. Researchers use external stimuli to induce people’s emotional responses, and use sensor technology to collect a variety of physiological indicators, including electroencephalography, ophthalmic electromyography, and skin conductance. These collected physiological signals are used as the basis for emotion recognition research and are validated as objective indicators of the true emotional state of humans. In particular, when emotional information is not easy to be directly conveyed or deliberately disguised, emotion recognition with the help of these physiological signals shows higher accuracy and efficiency. With the deepening of research, researchers are increasingly aware of the significant similarities and complementarities among various physiological signals. Therefore, an increasing number of researchers tend to combine EEG with other physiological indicators to carry out more in-depth research on emotion recognition.

Combining CNN and LSTM has significant advantages in emotion recognition tasks, as it can simultaneously capture the temporal and spatial information of EEG as well as the time series information of other signals, providing more comprehensive and in-depth information for emotion recognition [21]. Ben et al. [22] uses en-cod-decoder architecture to integrate electroencephalogram (EEG) and electromyogram (EMG) signals to efficiently reconstruct data from underlying representations, clearly revealing the mechanism of interaction between brain and muscle activity during emotion expression. Becker et al. [23] demonstrated the complementarity between different physiological signals by combining different physiological signals (EEG, skin EMG, respiration, and blood oxygen signals). The multimodal residual LSTM network proposed by Ma et al. [24] provides a new perspective and method for processing and analyzing multimodal physiological data. By sharing weights and residual connections, it is possible to learn correlations between different modalities and extract high-level features that are relevant to a particular task. Jia et al. [25] studied a Het-EmotionNet model that can simultaneously model the feature complementarity, correlation, and heterogeneity of multimodal data under a unified framework. Yilmaz et al. [26] extract core features by analyzing two-dimensional images of electroencephalogram (EEG) and electrooculogram (EOG) signals, which provide important input information for subsequent tasks such as emotion recognition. The characteristics of various modal information are individually extracted, followed by the extraction of inter-modal correlations using deep LSTM networks [27]. A multimodal emotion recognition model is optimized through feature selection [28], and a sentiment recognition model based on six physiological signals based on SFNN has been proposed [29]. Wang et al. fused the physiological features of EEG signals extracted by CNN and the visual features of facial expressions extracted by a pre-training network using the attention mechanism, which significantly improved the performance of emotion classification [30]. Cheng et al. proposed an innovative dynamic interactive network combined with a self-distillation mechanism for multimodal emotion recognition across subjects [31].

In summary, the application of multimodal fusion features overcomes the shortcomings of unimodality in the field of affective computing to a large extent. By integrating the differences and complementarities between different modalities, we can obtain more comprehensive and rich emotional information. The multimodal approach has obvious advantages in improving accuracy, enhancing robustness, and promoting cross-modal information fusion. This fusion of cross-modal information has opened up new development opportunities for affective computing, accelerating the rapid progress and widespread popularization of related technologies.

Although existing affective recognition has many advantages, existing methods still face two key challenges: the first is the granularity of feature representation, and existing methods often rely on single-scale feature extraction or separate global and local features. This processing method is difficult to fully capture the multi-scale information of EEG signals, resulting in some subtle but crucial features for emotion recognition being ignored. For example, when dealing with different emotions, due to the multi-scale morphology of EEG signals and different eye movements, single-scale feature representation often cannot accurately describe the physiological characteristics of emotions. The change in emotion-related physiological signals is a dynamic and complex process of time series, which contains temporal and spatial dependencies, and existing methods have shortcomings in modeling these dependencies. On the one hand, the modeling ability of long time series is limited, and it is difficult to capture long-term dependencies across multiple emotional stimulus cycles. On the other hand, the existing methods lack an adaptive weight allocation mechanism for the important characteristics of different signal regions. This means that the model cannot dynamically adjust the focus according to different emotional stimuli, thus affecting the accuracy of emotion recognition. These problems are particularly prominent in practical applications, which directly affect the stability and robustness of recognition performance.

Based on the limitations of current algorithms, this paper aims to improve the multi-scale modeling ability of multimodal features and the spatiotemporal dependent modeling effect. We combine multi-stream networks with a two-scale attention network (MSDSANet) feature representation to achieve more accurate and efficient multimodal emotion recognition. Through the effective integration of physiological information of different grain sizes, the richness and differentiation of feature representation were improved, and the adaptability of the model to complex emotional stimulation scenarios was enhanced. Compared with the prior art, it has a superior performance. The core results of this paper are summarized as follows:

(1) The MSDSANet model introduced in this paper consists of three core components: a multi-stream feature module, dual-scale attention module, and multimodal fusion module. These modules work together to form an efficient and comprehensive model, enabling the model to learn deep connections between multimodalities and classify emotions more reliably.
(2) In order to further enhance the recognition effect, MSDSANet adopts a multi-stream framework to combine the original EEG features with the spatiotemporal features of EEG in its 10–20 electrode system. Given the varying contributions of each EEG data stream to emotion recognition, it is imperative to investigate and exploit the complementarity among them to extract more diverse features.
(3) An attention-based multi-scale residual block is proposed to extract the features of 3D spatiotemporal matrix, and a lightweight multi-scale feature extraction structure is realized. In addition, the Efficient Channel Attention (ECA) strengthens the feature map extracted at each scale, eliminates redundant features, and improves the effectiveness of the features.
(4) MSDSANet uses multi-scale analysis techniques to accurately extract richer broadband spectral information from eye tracking signals, and effectively fuse spectral and spatial information at different scales. Subsequently, with the help of the dynamic weight allocation function of the attention mechanism, the fusion process of features of different scales was optimized, and the key information was highlighted, so as to improve the efficiency and quality of information integration.

## 2. MSDASNet Network Model Design

### 2.1. System Overview

In order to make better use of EEG and other signals to understand the characteristics of emotions in more detail, the MSDSANet model was proposed.

**Figure 1:**
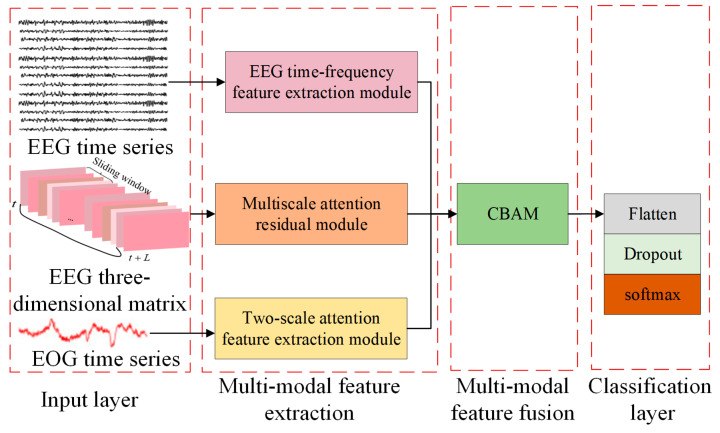
Overall architecture of the proposed MSDASNet model.

Emotions are complex and multidimensional concepts that involve many aspects, such as brain activity, physical reactions, and the external environment. For this reason, it is often difficult to fully and accurately grasp all the characteristics of emotions if they are limited to feature analysis using a single signal. To overcome this limitation, this paper proposes a multi-stream network and double-scale attention network (MSDSANet), which can integrate EEG signals and other signals (such as eye movement signals, EMG, skin conductance, etc.), and use the complementarity of these signals to reflect emotional states, thus comprehensively characterizing emotional states. The architecture of the model is shown in Figure 1, which includes three basic components: a multimodal feature extraction module, a multimodal feature fusion module, and a classification module.

Firstly, feature extraction methods for different modal data features are designed to ensure that emotion-related information can be captured effectively and accurately. For EEG data, a multi-stream network module is used to capture the core information from different dimensions, which can comprehensively extract and analyze the multidimensional information in the signal. For EOG data, an attention-based dual-scale module is introduced to extract richer shallow features. This module combines dual-scale analysis and attention mechanisms to capture more nuanced and important emotion-related information from EOG signals. Then, in the multimodal fusion stage, we used the Convolutional Block Attention Module (CBAM) attention to fuse dual-stream EEG features and ocular features and then directly used these fused multimodal features for classification tasks.

### 2.2. Attention-Based EEG Feature Extraction Module of Multi-Stream Network

This paper introduces a two-stream design for capturing more subtle and complex features from EEG signals. EEG has a complex structure and rich information, and the high-level semantic features in EEG can be extracted using the dual-stream module, which can effectively reflect the content and structure of EEG.

The positioning technology of scalp EEG provides EEG data with two key dimensions: the spatial dimension determined by the electrode layout and the temporal dimension formed by the change in electrode potential with time. In traditional methods, EEG data are mostly expressed by algorithms in the form of a two-dimensional matrix, where the shape of the matrix is determined by the number of channels, and the data from each sample point are organized into elements in the matrix. This three-dimensional representation not only maintains the temporal dimension information of the EEG data but also completely retains its spatial structure information. This information has been widely used in a variety of EEG classification tasks, and the accuracy and validity of the model have been significantly improved.

**Figure 2:**
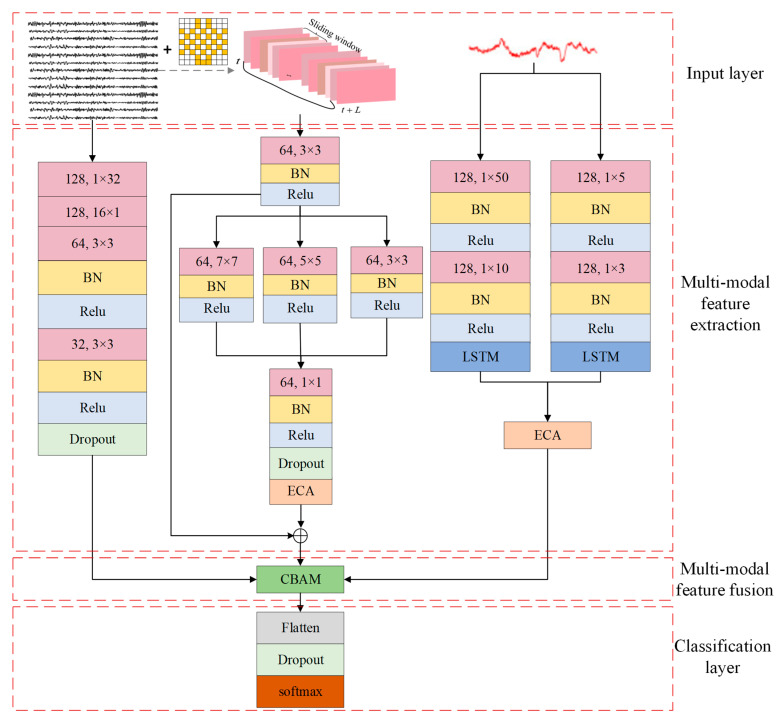
Multimodal emotion recognition model diagram of the MSDASNet network.

As shown in Figure 2, the MSDASNet proposed in this paper makes innovative use of a dual-stream network architecture, which carefully designs two core components to delve deeper into the characteristics of EEG signals. Specifically, the first core component is the EEG time-frequency feature extraction module, which focuses on accurately extracting time-frequency features from time series samples. The second core component is based on the multi-scale attention residual module, which is committed to efficiently collecting multi-scale time-frequency spatial information from the constructed 3D spatiotemporal matrix.

The original EEG time series retains the details of neural oscillations at the millisecond level, and the transient dynamic characteristics of δ (0.5–4 Hz), θ (4–8 Hz), α (8–13 Hz), β (13–30 Hz), γ (30–100 Hz) are completely preserved. However, traditional preprocessing methods, such as time-frequency transformation or spatial interpolation, may lead to the loss of phase-locking characteristics of event-related potential (ERP) in the time dimension. In addition, directly modeling the raw signal captures transient neural responses (such as the 300 ms peak latency of the P300 component). EEG is a dynamic and time-varying signal, not only the time series information of the signal is important, but also the spatial position information between the signals is equally important for emotion recognition. The role of different brain regions in emotion classification tasks is different, and the activation state of brain regions in the brain network changes dynamically with time during emotion induction. By constructing a three-dimensional spatiotemporal matrix of channel × time × space, we not only strictly maintain the topological constraint relationship of the electrode arrangement in the international 10–20 system mathematically, but also model the electrode distribution pattern closely related to emotional valence at the feature space level. The various regions of the EEG play different important roles in emotion regulation, and this spatially specific neural marker can only be effectively captured by maintaining the spatial relationship between electrodes.

The dual-flow architecture design of parallel processing EEG original time series and spatiotemporal topological matrix realizes the collaborative modeling of spatiotemporal dual attributes of neural information. The original time series processing flow accurately captures the dynamic characteristics of neural oscillations at the millisecond level, and the spatiotemporal topological matrix processing flow analyzes the spatial distribution pattern of cortical potential at the millimeter level, which overcomes the fundamental limitation of the traditional single-channel processing paradigm in terms of information completeness.

#### 2.2.1. EEG Time-Frequency Feature Extraction Module

Considering the multi-channel characteristics of EEG signals, a unique convolution strategy is designed for the time-frequency feature extraction module of the original time input. Specifically, the first convolution layer is subdivided into two independent convolution operations: the first convolution focuses on time-dimensional convolution processing for each individual electrode signal, aiming to capture timing features, followed by a second convolution that unfolds at all electrode or channel levels to integrate cross-channel information. In the subsequent step, we construct two continuous two-dimensional convolutional layers to process the EEG signals. Both convolution layers use a 3 × 3 convolution kernel and are set to 64 and 32 feature maps, respectively. To further improve the performance and stability of the network, we add a Batch Normalization (BN) layer and a ReLU activation function after each convolutional layer. This combination of structures can not only accelerate the training speed of the network but also greatly enhance the generalization ability of the model, enabling it to perform well when processing complex EEG data. In order to effectively prevent overfitting, this paper connects the dropout operation after activating the layer.

#### 2.2.2. MSARB: Spatiotemporal Multi-Scale Attention Residual Module

For the multi-scale attention residual block (MSARB) module, the input is a three-dimensional spatiotemporal matrix. This module aims to perform deep processing of the input 3D EEG spatiotemporal matrix by combining multi-scale features and attention mechanisms to extract richer and more discriminative features. Specifically, the module first uses a multi-scale convolution check input matrix to implement convolution operations to capture and extract spatiotemporal features at each scale. In this study, we modeled the hierarchical information processing mechanism of the nervous system to construct a multi-scale feature extraction system: high-frequency transient activities in the neighborhood of the 3 × 3 local receptive field focusing electrode were revealed to reveal the millisecond dynamic details of event-related potentials such as P300; it was further extended to a 5 × 5 scale receptive field to model cross-brain rhythm characteristics. The larger 7 × 7 macroscopic receptive field resolves the characteristics of the whole brain network.

Subsequently, the module incorporates Efficient Channel Attention (ECA) [32] to dynamically modulate the significance weights of features across various scales. By analyzing the interdependencies between channels, the weight of each channel is dynamically assigned by the ECA mechanism, and the weight of each channel is different so that the network can adjust itself to emphasize important features while reducing the impact of non-critical information. In addition, the introduction of residual connections is crucial to ensure the effective flow of information within the module, which significantly alleviates the gradient disappearance and gradient explosion problems that may be encountered in the training of deep neural networks, thus enhancing the learning efficiency and generalization performance of the network. Through this design, the multi-scale attention residual module can effectively extract features that contain both fine local information and global context from the 3D spatiotemporal matrix of EEG.

**Figure 3:**
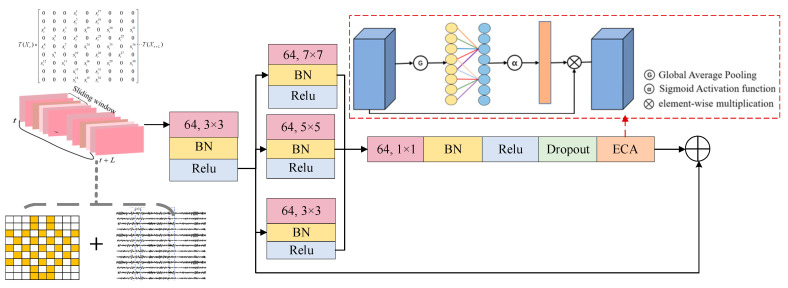
Multi-scale attention residual module MSARB model diagram.

As revealed in Figure 3, we use a two-dimensional convolution layer with a convolution window size of 3 × 3 to process the input data $h_{in}$ and capture relevant information from it:

(1) h0=ReBNμW3×3∗hin+b

where $\mu \left(\cdot \right)$ represents a convolutional layer, $BN\left(\cdot \right)$ represents a BN layer, Re⋅ represents a ReLU layer, $W_{3\times 3}$ represents the convolution weight, b represents the offset, where 3×3 is the kernel size, $h_{in}$ is the three-dimensional space-time matrix, and $h_{0}$ is the feature representation of the ReLU layer output.

Subsequently, three convolutional layers with different convolutional kernels (7×7, 5×5 and 3×3) are used to process the input data in parallel to extract features.

(2) hi=Re(BN(μ(Wi×i∗h0+bi)))

where i represents the different convolutional kernel sizes in the multi-scale residual block, which are 7×7, 5×5 and 3×3, $b_{i}$ represents the offset of the convolutional kernel i, and $h_{i}$ is the output feature of the ReLU layer.

Then, these three features are fused and expressed numerically as follows:

(3) $$h_{cat}=CAT(h_{7},h_{5},h_{3})$$

where $h_{cat}$ is the output feature of the concatenate layer, and CAT(⋅) stands for concat() operation.

Once the features are fused, they are processed using a 1 × 1 convolutional kernel.

(4) hcat1=Re(BN(μ(W1×1∗hcat+b)))

where $h_{cat1}$ is the output feature of the convolutional kernel 1×1, *BN*, and ReLU layers.

**Figure 4:**
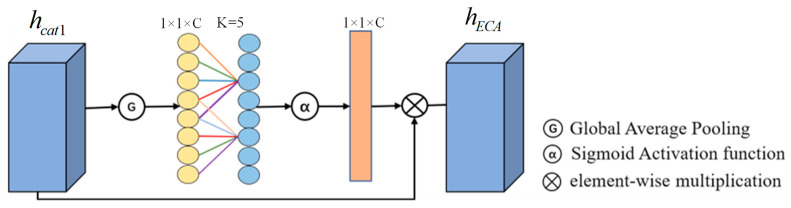
The flow chart of ECA attention mechanism.

As shown in Figure 4, the ECA mechanism was used to filter the fused features, and different weights were assigned to each feature. Firstly, the global average pooling of the fused feature graph is carried out to obtain a global feature representation of $G\left(\cdot \right)$. Then, based on the total number of input channels C and its logarithm, the preset parameters t and b calculate the adaptive 1D convolution kernel size k. The output of the one-dimensional convolution with a step size of 1 and kernel size k is calculated by the sigmoid activation function $\alpha \left(\cdot \right)$. Finally, the weights are multiplied with the input feature graphs $h_{cat1}$ by the broadcast mechanism to obtain feature graphs with different weights. Finally, the output feature diagram of ECA’s attention $h_{ECA}$ is obtained. The detailed calculation steps are as follows:

(5) $$k={\left|\frac{\log_{2}C}{t_{0}}+\frac{b1}{t_{0}}\right|}_{odd}$$

(6) $$G(h_{cat1})=\frac{1}{L}\sum_{j=1}^{L}h_{cat1j}$$

(7) $$h_{ECA}=\alpha (CONV1D_{k}(G(h_{cat1})))⊗h_{cat1}$$

where L is the length of each feature in the input feature map $h_{cat1}$. $\alpha \left(\cdot \right)$ is the sigmoid activation function, the constant b1 is assigned to 1, $t_{0}$ is assigned to 2, and ⊗ is the product of each element.

Finally, local skip connections are added to help the information flow, which is represented by Equation (8):

(8) $$h_{eeg2}=h_{ECA}+h_{0}$$

### 2.3. DSANet: Based on the Dual-Scale Attention Electroocular Feature Extraction Module

The size of the temporal convolutional kernel is critical for extracting frequency information from peripheral physiological signals, such as the electrooculogram signal EOG. Specifically, temporal convolution with large cores can efficiently capture lower frequencies and a wider range of frequency components, while temporal convolution with small cores is effective for extracting higher frequencies and finer frequency details. In order to make full use of the advantages of spatiotemporal convolution at different scales, we propose a dual-scale attention feature extraction module for peripheral physiological signals (such as EOG), which can accurately capture the multi-scale time-frequency information in the electrooculogram signals, thereby enhancing the comprehensiveness and depth of feature extraction. Subsequently, the ECA attention mechanism was used to intelligently fuse these features to further refine the feature set with high representativeness and discrimination.

Specifically, in this paper, a 1 × 50 long-window convolution kernel was used to capture macroscopic eye movement patterns (such as fixation duration and slow change trend of pupil diameter). The 1 × 5 short-time window convolutional kernel accurately extracts microscopic eye movement events (such as 20–30 ms transient pulses of micro-saccades and peak speed characteristics of rapid saccades), corresponding to the millisecond response characteristics of the visual cortex to sudden stimuli. The two-flow features are fused by ECA attention, in which the long-duration features dominate the modeling of slow-changing emotional states, while the short-duration features analyze the instantaneous physiological responses induced by emotions.

**Figure 5:**
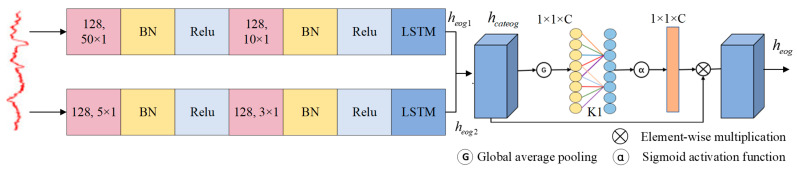
Multi-scale attention residual module DSANet model diagram.

As shown in Figure 5, in the first branching path, we focus on capturing broader spectrum and spatial information. First, we use a convolutional layer with a large convolution kernel of 1 × 50 to extract the low frequency features in the EOG signal. Subsequently, a convolution layer equipped with a 1 × 10 convolution kernel is used to further capture the time-frequency details of the feature map. Finally, we use a 768 cell LSTM layer to capture the temporal dynamic features in depth.

In the second branch path, we first apply a 1 × 5 convolution kernel to extract more detailed information from the EOG signal. Then, the spatiotemporal properties of the features are obtained through a convolutional layer equipped with a 1 × 3 convolution kernel. Finally, we deployed a 384-cell LSTM layer to analyze the temporal dimension of the feature map in depth.

Next, the ECA is used to intelligently fuse the two-branch features to further refine the feature set with high representativeness and discriminant power, and the specific process can be formalized as follows:

(9) $$h_{cateog}=CAT\left(h_{eog1},h_{eog2}\right)$$

(10) $$k1={\left|\frac{\log_{2}C1}{t_{1}}+\frac{b2}{t_{1}}\right|}_{odd}$$

(11) $$h_{cateog}=\frac{1}{L1}\sum_{j1=1}^{l1}h_{cateog_{j1}}$$

(12) $$h_{eog}=\alpha \left(CONV1D_{k1}\left(G\left(h_{cateog}\right)\right)\right)⊗h_{cateog}$$

where $h_{eog1}$ and $h_{eog2}$ are the outputs of the two branches of DSANet, based on the total number of input channels C1 and its logarithm, respectively, and the preset parameters t1 and b2 calculate the adaptive 1D convolution kernel size k1. The feature map $h_{cateog}\in {ℜ}^{C1\times L1}$ is output by passing the concatenate layer of $h_{eog1}$ and $h_{eog2}$, the constant b2 is assigned to 1, t1 is assigned to 2, $h_{eog}$ is the output feature map of ECA attention.

### 2.4. Attention-Based Multimodal Feature Fusion Module

**Figure 6:**
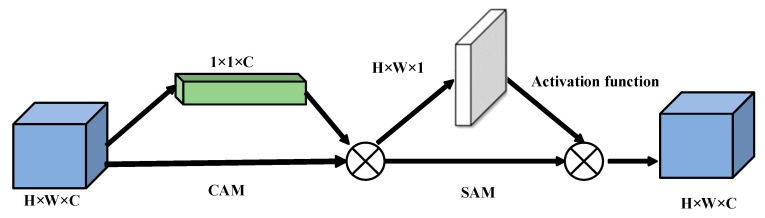
Schematic diagram of the CBAM attention block.

**Figure 7:**
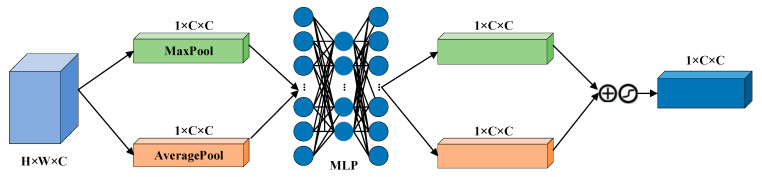
Schematic diagram of the channel attention module.

**Figure 8:**
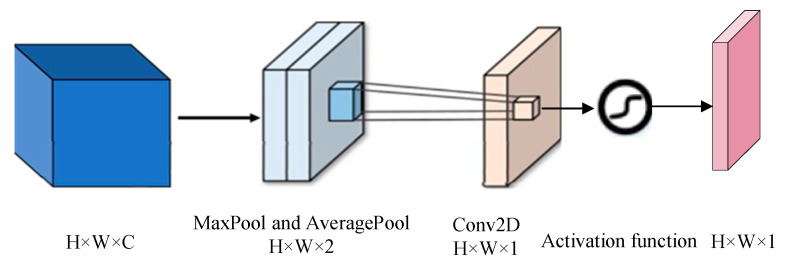
Schematic diagram of the space attention module.

This is example 1 of an equation: Different modal signals contain unique physiological features, and the fused features can promote the capture of emotional features more effectively. In order to improve the learning efficiency of multimodal features and ensure that they are more closely related to emotion recognition tasks within an adaptive framework, we introduce CBAM to merge the double-flow features from EEG with the double-scale features from EOG. The implementation diagram of the fusion module is shown in Figure 6, Figure 7 and Figure 8 [33].

The CBAM is divided into a Channel Attention Module (CAM) and a Space Attention Module (SAM). CAM enables neural networks to focus on information-rich feature channels while ignoring unimportant parts. In contrast, the SAM directs the network to focus on specific local regions of interest within the feature map. The whole process is as follows:

(13) $$h_{calall}=CAT(h_{eeg1},h_{eeg2},h_{eog})$$

where $h_{eeg1}$ is the output characteristic of the original timing input in the AMSNet module, and $h_{all}$ is the output feature map of the concatenate layer.

Given a feature graph $h_{all}$, CAM generates a one-dimensional channel attention vector $h_{ca}$ where each element represents the relative importance of the corresponding channel in the feature graph. At the same time, SAM also processes the input feature map to derive a three-dimensional spatial attention map $h_{all}$, which clearly shows which areas of attention information are particularly critical at different locations in the feature map. The running process is as follows:

(14) $$h_{ca}=f_{ca}(h_{catall})⊗h_{catall}$$

(15) $$h_{all}=f_{sa}(h_{ca})⊗h_{ca}$$

(16) $$f_{ca}(h_{calall})=\sigma (MLP(Avgpool(h_{calall}))+MLP(Maxpool(h_{calall})))$$

(17) $$f_{sa}(h_{ca})=\sigma (W_{7\times 7}(\left[Avgpool(h_{ca});Maxpool(h_{ca})\right]))$$

where $f_{ca}(h_{ca})$ is the channel attention weight, and $f_{sa}(h_{ca})$ is the space-level feature weight, Avgpool(⋅) and Maxpool(⋅) are the average and maximum pooling, respectively.

### 2.5. Classification Module

The features output by the multimodal feature fusion module will be fed into the classification module. The classification module comprises a Flatten layer, a Dropout layer, and a Dense layer. Specifically, the features are flattened in the Flatten layer, and the output feature map becomes a one-dimensional feature vector. Next, the final function output is passed to a fully connected layer containing the softmax activation function. This process generates predictive labels that correspond to each sample.

In the training process, cross entropy loss is adopted as a loss function to measure the error, which is defined in Equation (18):

(18) $$L=-\frac{1}{N}\sum_{i=1}^{N}y_{i}\log{\hat{y}}_{i}$$

where N is the total number of samples, and $y_{i}$ and ${\hat{y}}_{i}$ are the true and predicted labels of the *i*-th sample, respectively.

## 3. Experimental Setting

The proposed multimodal emotion recognition method was evaluated using the DEAP and DREAMER databases.

### 3.1. DEAP Databases

The DEAP dataset [34] covers the EEG activity and peripheral physiological responses of 32 participants while watching music videos. In this paper, a preprocessed version of the dataset is used, and each EEG record consists of two parts: a 3-s resting baseline signal (acquired in a relaxed state) and a 60-s experimental signal (acquired while watching a video). After watching each video, the participants were asked to rate their arousal, valence, liking, and dominance on a scale of 1 to 9 based on their feelings. Therefore, the size of the resulting label matrix is 40 rows and 4 columns, corresponding to 40 trials and 4 evaluation labels (valence, arousal, dominance, liking).

### 3.2. DREAMER Databases

The DREAMER dataset [35] contains EEG and ECG data from 23 participants, which were collected while they watched movie clips on 18 different emotional topics. For each participant, EEG signals were recorded using 14 electrodes at a sampling frequency of 128 Hz, and ECG signals were collected using two electrodes at a sampling frequency of 256 Hz. During data processing, the EEG signal was preprocessed by band-pass filtering, and artifact subspace reconstruction (ASR) was applied. The complete dataset for each participant was eventually integrated into the following three parts: first, 18 segments of baseline EEG and ECG signals acquired in a relaxed state were used to establish a reference for the individual’s resting state, followed by 18 EEG and ECG signal segments recorded in the experimental context, that is, when watching a specific emotional movie clip, which reflected changes in brain activity and cardiac heart signals under emotional stimuli. Finally, there were 18 matching sets of labels, each of which contained scores on three dimensions: valence, arousal, and dominance, which provided subjective quantitative feedback on the viewing experience of the participants.

### 3.3. Experimental Data and Processing

In order to highlight EEG features directly related to the experimental stimulus and to more accurately analyze the changes in brain activity under experimental conditions, we used the same data preprocessing method described in a previous paper [36], which uses a one second sliding window to segment non-baseline signals to expand the number of EEG samples.

In the DEAP dataset, each experimental signal that has been preprocessed is further segmented through a one second sliding window. Given that each DEAP participant participated in 40 experiments and each experimental signal contained 60 s of valid data (after removing the baseline signal), approximately 2400 samples could be generated per participant (40 trials multiplied by one window per second, i.e., 60 fragments). In the DEAP dataset, each EEG sample is recorded at a sampling rate of 128 Hz and contains 32 data channels, resulting in a matrix of 32 rows and 128 columns.

In contrast, each experimental signal in the DREAMER database is not uniform in length, and its length is determined by the length of the film clips being watched. However, by using the same one second sliding window technique, each participant in the dreamer dataset was still able to generate a certain number of EEG samples. Specifically, after window segmentation, each participant obtained 3728 EEG samples from the DREAMER dataset. Since the dataset was recorded using 14 EEG electrodes and each EEG sample was sampled at 128 Hz, each EEG sample was represented as a matrix of 14 rows and 128 columns.

When segmenting the signals using the sliding window technique, we ensured that each segment of the signal matched its corresponding label so that the EEG signal could be accurately correlated with emotion evaluation in subsequent analyses. For the DEAP dataset, dividing the score range of labels (arousal, valence, and dominance) from 1 to 9, we set a threshold of 5 to distinguish between high and low levels. Specifically, a label score greater than 5 is considered to indicate a high level (high arousal/high valence); conversely, if the score does not exceed 5, it is considered low (low arousal/low valence). This division allows us to treat continuous affective evaluation data in a binary manner. Similarly, in the DREAMER dataset, we use a similar labeling method. In order to effectively distinguish between the different levels, we determine 3 as the critical value.

### 3.4. Evaluation Metrics

The evaluation indicators of emotion recognition used in this paper include classification accuracy, kappa coefficient, F1 score, sensitivity (SN), and specificity (SP).

#### 3.4.1. Accuracy

Accuracy is used as a measure to evaluate the EEG classification, which is calculated as follows:

(19) $$acc=\frac{A}{Num}$$

where A refers to the number of samples correctly classified by the algorithm, and Num refers to the total number of samples to be classified.

#### 3.4.2. Kappa Coefficient

Kappa coefficient is usually used to measure the classification accuracy of emotion recognition tasks, which can eliminate the influence of random samples on classification accuracy. The formula for calculating the kappa coefficient is Equation (20).

(20) $$kappa=\frac{p-p_{e}}{1-p_{e}}$$

where p is the total classification accuracy, and $p_{e}$ refers to the ratio of theoretical consistency, and the random identification accuracy under random samples. If there are N categories of EEG signals, then the random identification accuracy is $\frac{1}{N}$, and the kappa coefficient can be expressed as follows:

(21) $$Kappa=\frac{p-1/N}{1-1/N}=\frac{Np-1}{N-1}$$

#### 3.4.3. F1 Score, SN and SP

f1 score, SN and SP are calculated as follows:

(22) $$f1=2\times \frac{P\times SN}{P+SN}$$

(23) $$SN=\frac{TP}{TP+FN}$$

(24) $$SP=\frac{TN}{TN+FP}$$

(25) $$P=\frac{TP}{\left(TP+FP\right)}$$

where TP, FN, FP, and TN represent the number of true positives, false negatives, false positives, and true negatives, respectively, and P stands for accuracy.

## 4. Experimental Results

### 4.1. Experimental Design

In the experiment, we used the Keras framework to train the model. For each participant, we calculated their average accuracy over 10 cross-validations [37] as the final result. Subsequently, we aggregated the average accuracy of all participants to obtain an overall average accuracy rate, which was used as the final measure of the method performance. In terms of model training, we used different parameter settings for different datasets. For the DEAP dataset, we employed a random gradient descent method (SGD) optimizer to minimize the marginal loss function, where the learning rate was set to $10^{-5}$, the batch size was set to 64, and the number of training epochs was set to 40. This parameter configuration was designed to balance the training speed and convergence performance of the model. For the DREAMER dataset, we continued with the SGD optimizer and maintained the learning rate at $10^{-5}$, the batch size was still set to 64, but the number of training cycles was adjusted appropriately to 30. In all experiments, we ensured random scrambling of the samples to eliminate the possible impact of the data order on the model training.

### 4.2. The Results of the DEAP Database

In this chapter, the following comparative methods are used: Conti-CNN [38], CNN- RNN [39], DGCNN [15], ACRNN [40], MLF-CapsNet [36], GcForest [41], 3DFR-DFCN [42], AP-CapsNet [43], GLFANet [44] and ICaps-ResLSTM [45]. Table 1 presents the average accuracy of the 32 subjects in three dimensions when performing emotion recognition tasks on the DEAP dataset.

The experimental results show that our model outperforms all the other models in arousal, valence, and advantage. Specifically, the accuracy of the model is as high as 98.07% on the arousal dimension, 98.19% on the valence dimension, and 98.24% on the dominance dimension. It is worth mentioning that compared with the model with the worst performance in each dimension, the accuracy of our model improved by 8.62% in the arousal dimension, 7.95% in the valence dimension, and 7.99% in the dominance dimension. This series of data strongly proves the effectiveness and superiority of our proposed method in the emotion recognition task based on the DEAP database.

### 4.3. The Results of the DREAMER Database

Table 2 shows the average accuracy of the three dimensions obtained by 23 subjects in the DREAMER dataset when performing the emotion recognition task. The experimental results show that our model outperforms all the other models in arousal, valence, and dominance. Specifically, the accuracy of the model is as high as 94.83% on the arousal dimension, 95.34% on the valence dimension, and 95.25% on the dominance dimension. It is worth emphasizing that compared with the model with the weakest performance in all dimensions, our model achieved a 14.9% improvement in accuracy in the arousal dimension, a 13.82% improvement in the valence dimension, and a significant increase of 14.31% in the dominance dimension. Compared to the most advanced ICaps-ResLSTM, our method has improved classification accuracy by about 0.12%, 0.37%, and 0.29% on average across the three dimensions. This series of data strongly demonstrates that our model shows significant and comprehensive advantages in the EEG emotion recognition task using the DREAMER database.

According to the statistical results in Table 1 and Table 2, *p*_values1, *p*_values2, and *p*_values3 represent the *p*-values of the significant difference between the average accuracy rate of the method in this paper and that of several comparison methods in valence, arousal, and dominance of each subject, respectively. These *p*-values were calculated using a *t*-test to evaluate whether there were significant differences in classification performance between the proposed method and the comparison method in different affective dimensions.

It can be seen from Table 2 that in most cases, the *p*-value between the method in this paper and that in arousal and dominance is less than 0.05, indicating that the classification performance of the method in this paper is significantly better than that of the comparison method in valence, for arousal and dominance. However, on the DEAP dataset, although the method showed significant differences compared to most existing algorithms (*p* < 0.05), no significant differences were found with the ICaps-ResLSTM algorithm. On the DREAMER dataset, there is no significant difference between the proposed method and the MLF-CapsNet, GLFANet, and ICaps-ResLSTM algorithms. This phenomenon is mainly due to the similarity between the proposed algorithm and the ICaps-ResLSTM algorithm in the recognition results for some subjects. For example, in the DEAP data set, the average accuracy of the ten-fold cross-validation of subjects 3, 11, and 13 was 0.9917, 0.9708, and 0.9954, respectively. The corresponding results of ICaps-ResLSTM were 0.9832, 0.9694, and 0.9854, with little difference in accuracy between them. This overlapping or equivalent performance results in the absence of significant differences in the statistical analysis.

Although the difference between the proposed method and ICaps-ResLSTM in performance dominance is not significant, combining the experimental results of all subjects and arousal dimensions (valence, arousal, and dominance), the proposed method shows a higher classification accuracy in most cases. In addition, combined with the comparison results of computational efficiency and cost in Table 3, the training efficiency of the proposed method is significantly better than that of the comparison method (such as ICaps-ResLSTM). While ICaps-ResLSTM improves accuracy by increasing model complexity, it also significantly increases computational costs and resource consumption. In contrast, the proposed method achieves a balance between model performance and computational resource consumption and reduces computational complexity while ensuring high classification accuracy.

### 4.4. Network Visualization

**Figure 9:**
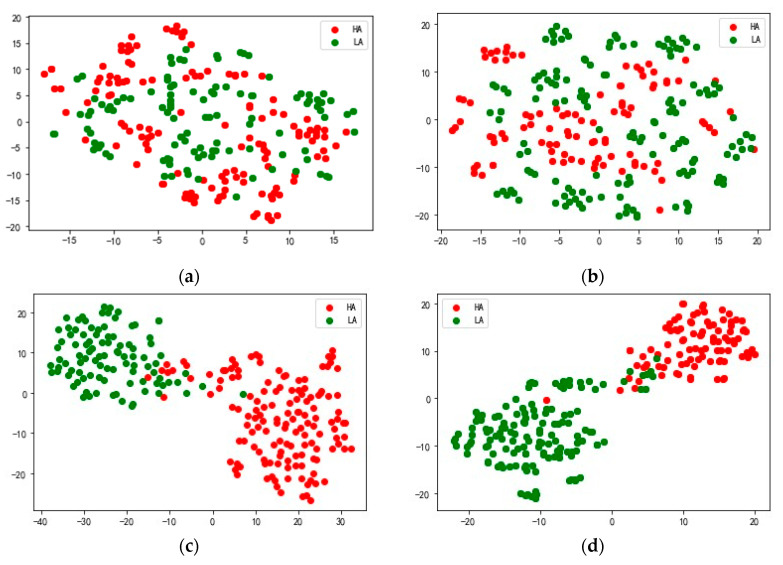
Visualization function of the MSDSANet model at different levels on the DEAP data set. (**a**) shows the original feature distribution of the arousal dimension of subject S23 in the DEAP data set, (**b**) the feature distribution of the HA and LA output by the model DSANet, (**c**) the feature distribution of the HA and LA output by the model MSARB, and (**d**) the feature distribution of the HA and LA output by the model CBAM layer.

In order to better understand the feature extraction ability of the model, we use the nonlinear dimension reduction algorithm t-SNE [46] to visualize the extracted features. Taking S23 in the DEAP data set and 20 in the DREAMER data set as examples, the high-dimensional features extracted from the attention fusion layer of the test data are mapped to a two-dimensional space, and the results are shown in Figure 9. The red dots represent feature data corresponding to high arousal (HA), and the green dots represent feature data corresponding to low arousal (LA). Figure 9a shows the original feature distribution of the arousal dimension of subject S23 in the DEAP data set, Figure 9b the feature distribution of the HA and LA output by the model DSANet, Figure 9c the feature distribution of the HA and LA output by the model MSARB and Figure 9d the feature distribution of the HA and LA output by the model CBAM layer.

**Figure 10:**
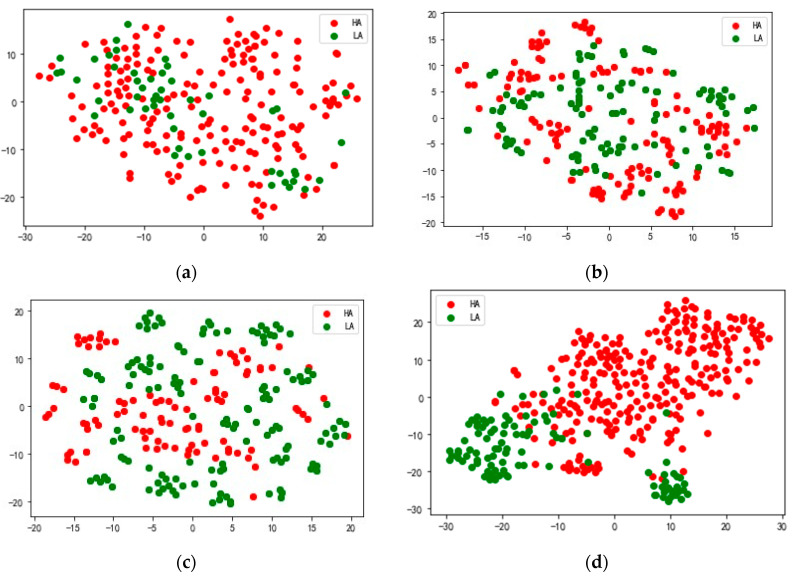
Visualization function of the MSDSANet model at different levels on the DERAMER data set. (**a**) shows the original feature distribution of the arousal dimension of subject 20 in the DERAMER data set, (**b**) the feature distribution of the HA and LA output by the model DSANet, (**c**) the feature distribution of the HA and LA output by the model MSARB, and (**d**) the feature distribution of the HA and LA output by the model CBAM layer.

Figure 10a shows the feature distribution of the arousal dimension of subject 20 in the DREAMER data set, Figure 10b the feature distribution of HA and LA output by model DSANet, Figure 10c the feature distribution of HA and LA output by model MSARB, and Figure 10d the feature distribution of HA and LA output by model CBAM layer. The results show that in the high-dimensional features extracted from the CBAM layer of the model, the overlapping area between the HA and LA is significantly reduced, the inter-class separation degree is significantly improved, and the classification boundary is clearer. These results fully prove the superiority of our model in multimodal feature fusion and generalization, which can effectively extract discriminative emotional features and provide reliable feature representation for emotional recognition tasks.

### 4.5. Ablation Test

In order to verify the effectiveness of the MSDSANet model in improving classification accuracy, this section conducts valence classification experiments on the DEAP and the DREAMER datasets, systematically evaluating the role of each component of the model. The MSDSANet method integrates three core components: MSARB (multi-scale attention residual block), DSANet (two-scale attention network), and multimodal feature fusion module. Among them, MSARB represents a two-stream EEG feature extraction model that integrates the original EEG time series and three-dimensional EEG matrix as inputs; MSARB1 represents an EEG feature extraction model using only the original EEG time series input; and MSARB2 represents an EEG feature extraction model using only the three-dimensional EEG matrix input. DSANet represents an eye electrical feature extraction model integrating 1 × 50 large convolutional nuclei and 1 × 5 small convolutional nuclei; DSANet1 represents an eye electrical feature extraction model using only 1 × 50 large convolutional nuclei; DSANet2 represents an eye electrical feature extraction model using only 1 × 5 small convolutional nuclei. To fully analyze the contribution of each module, we designed the following comparison model: Model1 (single-mode EEG model, using only MSARB), Model2 (single-mode EOG model, Only DSANet is used), Model3 (multimodal model without 3D matrix input branches), Model4 (multimodal model without original EEG time series input branches), Model5 (multimodal model without 1 × 5 small convolutional kernel eye electrical feature extraction branches), Model6 (no 1 × 50 large convolutional kernel eye electrical feature extraction branches) Modal model).

First, experimental data showed that the ablation results in the DEAP database are shown in Table 4. By comparing Model 1, Model 2, and our model, we found that the introduction of multimodal modules significantly improved the accuracy of the valence dimension by 1.24% and 2.05%, respectively, and the kappa coefficient by 3.02% and 3.95%, respectively. This result shows that the model can obtain richer and more comprehensive data features through multimodal fusion, thus improving the accuracy and robustness of the model.

Experimental data show that our model is superior to Model 3 and Model 4 in accuracy and F1 value. Specifically, the MSARB two-flow structure adopted by our model shows clear advantages in this task over raw EEG data input alone and three-dimensional matrix input alone.

In addition, when we add DSANet to the model, the increment of accuracy, kappa value, and F1 value of the larger convolutional electroocular feature single branch in the valence dimension is 0.61%, 1.27%, and 0.74%, respectively. The increments of accuracy, kappa value, and F1 value in the valence dimension of the small convolutional electroocular feature single branch are 0.46%, 0.36%, and 0.19%, respectively. Although the performance improvement is not obvious, this finding also highlights that the dual-scale ophthalmic feature extraction module can provide more information for the model to further improve the recognition performance. These results prove the superiority of our model in the emotion recognition task of the DEAP count set.

Similar to the ablation results of the DEAP dataset in Table 4, the ablation results of the DREAMER dataset are presented in Table 5. After removing the EEG feature extraction module and EOG(or ECG) feature extraction module, the accuracy, kappa, f1, SN, and SP indexes of Model 1 and Model 2 showed a decreasing trend compared with our model in the titer dimension. The results show that multimodal features play an important role in maintaining and improving the recognition performance of our model. Compared with Model 3, the accuracy of our model is increased by 0.51%, 1.43%, and 0.66% in kappa and f1, respectively; compared with Model 4, the increment of MSDSANet is 2.20%, 7.95%, and 3.77%, respectively, indicating that EEG multi-flow structure can improve the comprehensiveness and accuracy of model feature extraction. After the removal of the large-scale electrical feature extraction module, the index accuracy, kappa, and f1 scores of Model 5 decreased by 0.22%, 0.31%, and 1.19%, respectively; after the removal of the small-scale electrical feature extraction module, the three indexes of Model 6 decreased by 0.52%, 2.63%, and 1.24%, respectively.

In summary, the ablation experiment results show that each module contributes to improving the recognition ability of the model, and a more accurate and robust recognition model can be obtained by combining different module designs.

### 4.6. Robustness Analysis Under Noise Conditions

In order to verify the robustness and reliability of our model in real scenes, we systematically evaluate the performance of the model in different SNR environments to simulate various practical application scenarios from laboratory conditions to extreme noise environments. Three typical noise levels are selected for the experiment: SNR = 10 dB (mild interference environment), SNR = 0 dB (typical real scene) and SNR = −5 dB (extreme interference environment). No noise represents the performance of recognition using data sets without noise.

In the valence dimension emotion recognition of the DEAP data set and DREAMER data set, we add Gaussian white noise of different intensities to the original EEG signal, and use the preprocessed data to train and test the model. The experimental results are presented in Table 6. Our model shows remarkable robustness under noisy conditions: when SNR = 10 dB, the classification accuracy of the DEAP data set and DREAMER data set reaches 94.58% and 90.05%, and when SNR = 0 dB, the accuracy remains at 85.71% and 84.95%, respectively, even under an extreme noise environment (SNR = −5 dB). The accuracy of the DEAP dataset remained at 83.75%, a performance decrease of about 14% compared to the noise-free condition. The DREAMER dataset maintained an accuracy rate of 79.3%, a performance decrease of about 16.38% over the noise-free condition. These results show that our model can maintain a stable performance under different noise levels and exhibits good environmental adaptability.

## 5. Conclusions

In this paper, a multimodal emotion recognition model is proposed to address the challenges of inaccurate feature representation and inadequate spatiotemporal dependence modeling in EEG emotion recognition. In this model, the AMSNet structure was introduced to realize the integration of multi-stream network features, and the MSARB was adopted to promote the integration of multi-scale features. In addition, DSANet was used to accurately capture multi-scale time-frequency information in the electric eye signal, thus enhancing the comprehensiveness and depth of feature extraction. The recognition performance of this model on two commonly used data sets exceeds that of existing methods and shows good robustness and generalization ability.

Most existing emotion recognition methods are based on well-designed deep network structures, and the utilization and enhancement of the data is not sufficient. In the future, we will focus on developing more efficient data enhancement strategies to further improve the model performance and generalization. Secondly, current research on EEG emotion mainly focuses on closed experimental scenarios, while in practical application scenarios, such as classrooms, driving, and other environments, the adaptability of the model needs to be improved.

## Figures and Tables

**Table 1:** Average accuracy and standard deviation n of different methods on the DEAP database (%).

Method	Valence	Arousal	Domiance	*p*-Vaule 1	*p*-Vaule 2	*p*-Vaule 3
Conti-CNN	89.45	90.24	90.25	*p* < 0.05	*p* < 0.05	*p* < 0.05
CNN−RNN	89.92	90.81	90.90	*p* < 0.05	*p* < 0.05	*p* < 0.05
DGCNN	92.55	93.50	93.50	*p* < 0.05	*p* < 0.05	*p* < 0.05
gcForest	97.69	97.53	97.62	*p* < 0.05	*p* < 0.05	*p* < 0.05
AP-CapsNet	93.89	95.04	95.08	*p* < 0.05	*p* < 0.05	*p* < 0.05
MLF-CapsNet	96.69	96.84	97.73	*p* < 0.05	*p* < 0.05	*p* > 0.05
GLFANet	94.53	94.91	95.35	*p* < 0.05	*p* < 0.05	*p* < 0.05
3DFR-DFCN	95.32	94.59	94.78	*p* < 0.05	*p* < 0.05	*p* < 0.05
ICaps-ResLSTM	97.94	98.06	98.15	*p* > 0.05	*p* > 0.05	*p* > 0.05
MSDSANet (ours)	98.07	98.19	98.24	-	-	-

**Table 2:** Average accuracy and standard deviation n of different methods on the DREAMER database (%).

Method	Valence	Arousal	Domiance	*p*-Vaule 1	*p*-Vaule 2	*p*-Vaule 3
Conti-CNN	84.54	84.84	85.05	*p* < 0.05	*p* < 0.05	*p* < 0.05
CNN−RNN	79.93	81.48	80.94	*p* < 0.05	*p* < 0.05	*p* < 0.05
DGCNN	89.59	88.93	88.63	*p* < 0.05	*p* < 0.05	*p* < 0.05
ACRNN	93.71	94.03	94.11	*p* < 0.05	*p* < 0.05	*p* < 0.05
gcForest	89.03	90.41	89.89	*p* < 0.05	*p* < 0.05	*p* < 0.05
MLF-CapsNet	94.59	95.26	95.13	*p* > 0.05	*p* > 0.05	*p* > 0.05
GLFANet	94.57	94.82	94.51	*p* > 0.05	*p* > 0.05	*p* > 0.05
3DFR-DFCN	93.15	91.30	92.04	*p* < 0.05	*p* < 0.05	*p* < 0.05
ICaps-ResLSTM	94.71	94.97	94.96	*p* > 0.05	*p* > 0.05	*p* > 0.05
MSDSANet (ours)	94.83	95.54	95.25	-	-	-

**Table 3:** Time-consuming comparisons.

Model	DEAP		DREAMER	
	Train	Test	Train	Test
	Time/s	Time/ms	Time/s	Time/ms
Conti-CNN	12.6405	0.1141	20.6040	0.0767
CNN–RNN	656.3955	1.0554	602.0744	0.9210
DGCNN	7.0225	0.3208	10.2529	0.1820
MLF-CapsNet	1338.3379	48.2138	635.9729	14.8009
SFCSAN	568.659	20.346	243.873	15.872
gcForest	693.4861	10.5672	1307.406	7.4973
3DFR-DFCN	524.3741	11.0211	1143.527	6.1321
ICaps-ResLSTM	1018.267	43.376	814.327	26.465
MSDSANet	606.7732	10.3793	379.7394	8.3927

**Table 4:** Experimental results of the ablation of the DEAP dataset. (The symbol “√” indicates the use of a module, while “×” denotes the non-use of the module.)

Models	MSARB	MSARB1	MSARB2	DSANet	DSANet1	DSANet2	Acc	Kappa	F1	SN	SP
Model1	√	×	×	×	×	×	0.9685	0.9306	0.9653	0.9759	0.9742
Model2	×	×	×	√	×	×	0.9417	0.8829	0.9414	0.9767	0.9627
Model3	×	√	×	√	×	×	0.9708	0.9404	0.9702	0.9852	0.9519
Model4	×	×	√	√	×	×	0.9691	0.9313	0.9662	0.9774	0.9726
Model5	√	×	×	×	√	×	0.9747	0.9474	0.9727	0.9766	0.9696
Model6	√	×	×	×	×	√	0.9762	0.9561	0.9781	0.9796	0.9785
MSDSANet	√	×	×	√	×	×	0.9807	0.9596	0.9800	0.9827	0.9760

**Table 5:** Experimental results of the ablation of the DREAME dataset. (The symbol “√” indicates the use of a module, while “×” denotes the non-use of the module.)

Models	MSARB	MSARB1	MSARB2	DSANet	DSANet1	DSANet2	Acc	Kappa	F1	SN	SP
Model1	√	×	×	×	×	×	0.9409	0.8658	0.9329	0.9560	0.9098
Model2	×	×	×	√	×	×	0.8575	0.6851	0.8419	0.9356	0.7266
Model 3	×	√	×	√	×	×	0.9435	0.8869	0.9435	0.9337	0.9545
Model4	×	×	√	√	×	×	0.9274	0.8283	0.9140	0.9762	0.8250
Model 5	√	×	×	×	√	×	0.9462	0.8970	0.9385	0.9680	0.9316
Model6	√	×	×	×	×	√	0.9434	0.8761	0.9380	0.9436	0.9310
MSDSANet	√	×	×	√	×	×	0.9483	0.8998	0.9498	0.9793	0.9339

**Table 6:** The recognition performance of MSDSANet under noise conditions.

Valence	Models	Acc	Kappa	F1	SN	SP
DEAP	−5 dB	0.8375	0.6720	0.8360	0.8615	0.8019
	0 dB	0.8751	0.7482	0.8739	0.8593	0.8952
	10 dB	0.9458	0.8908	0.9454	0.9466	0.9450
	No noise	0.9807	0.9596	0.9800	0.9827	0.9760
DREAMER	−5 dB	0.7930	0.5592	0.7792	0.6871	0.8622
	0 dB	0.8495	0.6837	0.8418	0.8924	0.7852
	10 dB	0.9005	0.7918	0.8958	0.9014	0.9000
	No noise	0.9483	0.8998	0.9498	0.9793	0.9339

## Data Availability

The datasets that support the experimentation work of this paper are available through controlled access. DEAP Dataset: https://www.eecs.qmul.ac.uk/mmv/datasets/deap/download.html (accessed on 12 August 2019) and DREAMER Dataset: https://zenodo.org/records/546113 (accessed on 21 July 2022).

## Abbreviations

The following abbreviations are used in this manuscript:

MSDSANet	Multi-stream network and dual-scale attention network
MSARB	Multi-scale attention residual block
DSANet	Dual-scale attention network
ECA	Efficient Channel Attention
CBAM	Convolutional Block Attention Module
EEG	Electroencephalogram
EOG	Electrooculogram
ECG	Electrocardiogram
